# Sequential and Dynamic Variations of IL-6, CD18, ICAM, TNF-*α*, and Microstructure in the Early Stage of Diabetic Retinopathy

**DOI:** 10.1155/2022/1946104

**Published:** 2022-01-27

**Authors:** Ang Xiao, HuiFeng Zhong, Lei Xiong, Lin Yang, YunFang Xu, SiMin Wen, Yi Shao, Qiong Zhou

**Affiliations:** ^1^Department of Ophthalmology, The First Affiliated Hospital of Nanchang University, Jiangxi Province Ocular Disease Clinical Research Center, Nanchang, 330006 Jiangxi, China; ^2^Departments of Intensive Care, The First Affiliated Hospital of Gannan Medical University, Ganzhou, 341000 Jiangxi, China; ^3^Department of Pathology, The First Affiliated Hospital of Nanchang University, Nanchang, 330006 Jiangxi, China

## Abstract

**Objective:**

The purpose of this project is to make sequential and indepth observation of the variations of retinal microvascular, microstructure, and inflammatory mediators at the early stage of diabetic retinopathy (DR) in streptozotocin-induced diabetes mellitus (DM) rats.

**Methods:**

DM was induced by a single intraperitoneal injection of 60 mg/kg body weight streptozotocin (STZ). The fluorescein fundus angiography, hematoxylin and eosin staining, periodic acid-Schiff staining, fluorescence imaging techniques, quantitative real-time PCR, and vascular endothelial growth factor- (VEGF-) A ELISA were performed on the 8^th^ day, at the 4^th^ week, 6^th^ week, 8^th^ week, and 10^th^ week after DM induction, respectively.

**Results:**

In this study, we observed not only the decrease of retinal ganglion cells (RGCs) and the increase of endotheliocytes to pericytes (E/P) ratio, acellular capillaries, and type IV collagen-positive strands began to occur on the 8^th^ day after induction but the vascular permeability and new vessel buds began to appear in the diabetes group at the 8^th^ week, while the expression of VEGF-A, VEGF mRNA, IL-6 mRNA, ICAM mRNA, and TNF-*α* mRNA were significantly higher in the diabetes group compared with the normal group(*P* < 0.01) on the 8^th^ day after induction and maintained a high expression level throughout the 10-week observation period. However, the expression of CD18 mRNA began to increase significantly at the 4^th^ week after induction and reached a peak at the 6^th^ week.

**Conclusion:**

Our study indicated the abnormal alterations of microvessels, microstructure, and inflammatory mediators at the early stage of DR, which confirms and supplements the previous research, and also promotes an indepth understanding and exploration of the pathophysiology and underlying pathogenesis of DR.

## 1. Introduction

Diabetic retinopathy (DR) remains a leading cause of vision loss in the working age population of industrialized regions [1]. A third of the 463 million people with diabetes [2] have signs of diabetic retinopathy, and a third of these might suffer from severe retinopathy or macular edema [3]. DR is categorized as nonproliferative DR (NPDR) and proliferative DR (PDR). Due to the fact that the patients without typically asymptomatic, NPDR (especially the mild NPDR) usually represents the early stage of DR. Early diabetic retinopathy is mainly characterized by loss of pericytes and retinal ganglion cells (RGCs), overexpression of vascular endothelial growth factor (VEGF), and compensatory synthesis and deposition of extracellular proteins. In addition, a variety of inflammatory mediators on the retina can be upregulated in the early stage of diabetes, including intercellular adhesion molecule-1 (ICAM-1), VEGF, nuclear factor kappa B (NF-*κ*B), inducible nitric oxide synthase (iNOS), tumor necrosis factor (TNF)-*α*, CD18, and local inflammatory response playing an important role in the occurrence and development of DR [4, 5].

Researchers have conducted some studies on early pathological changes, occurrence, and development of DR. One study [6] indicated that RGC apoptosis increased at the 4^th^ week of the diabetes course, and that this early abnormality of neurons may be due to the loss of nerve cells. Moreover, Kuwabara et al. [7] pointed out that microvascular pericytes were lost in the early stage of diabetic retinal tissue, and that the loss of pericytes in the vascular wall could lead to the formation of microhemangioma. Based on a study by Ayalasomayajula et al. [8], the vitreous VEGF expression of diabetic rats was significantly higher than that of normal control group on the 8^th^ day after induction. In the 4-week observation period, the VEGF expression in vitreous of diabetic rats was the highest at the 4^th^ week, which was significantly higher than that of the normal control group [9]. The overexpression of VEGF will transform or alter the occurrence of neovascularization and improve vascular permeability, leading to retinal structural and functional abnormalities [10].

**Figure 1:**
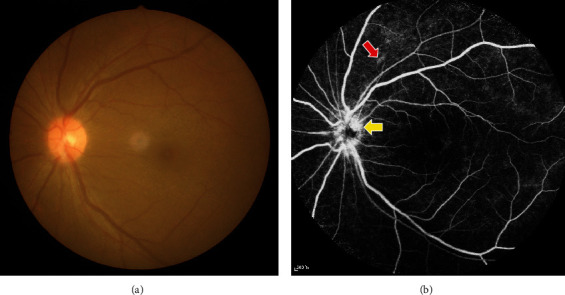
Example of the early stage of DR patients was examined on fundus camera (a) and fluorescence fundus angiography (b). (a) Fundus photography showed no obvious abnormalities. (b) Optic disc capillaries showed dilation and leakage (yellow arrow) and hemangioma capillanisum (red arrow).

Although the studies above have investigated the pathological changes of retinal structure in the early stage of DR, they have their own limitations, such as discontinuities in time and differences in results. The pathological changes of retinal structure in the early stage of DR at continuous time nodes remain unclear, and the theoretical basis for the early stage of DR needs further research. To further clarify the retinal microvascular injury, microstructure changes, and expression of interleukin-6 (IL-6), CD18, ICAM, TNF-*α* and VEGF in the early stage of DR (Figure 1), we conduct sequential and indepth observation of the occurrence and development at the early stage of DR in diabetic rats, especially using fluorescein fundus angiography (FFA) to examine the fundus of living organisms.

## 2. Material and Methods

### 2.1. Animals

A total of 50 eight-week-old male Sprague-Dawley (SD) rats (weight, 295 ± 15 g) were purchased from the Animal Center of Nanchang University (Nanchang, China). Rats were maintained under a condition of controlled temperature (23 ± 2°C), humidity (50%), and lighting (12-h light/dark cycle) and had ad libitum access to sterilized standard laboratory chow and water. Rats were treated in accordance with principles of animal ethics and were anesthetized with an intraperitoneal injection of pentobarbital (40 mg/kg body weight; Sigma-Aldrich; Merck Millipore, Darmstadt, Germany) for the subsequent experiments. All experiments were conducted in accordance with the Instruction and Administration of Experimental Animals and were approved by the Medical Ethics Committee of the First Affiliated Hospital of Nanchang University.

### 2.2. Diabetes Induction and Experimental Groups

Diabetes mellitus (DM) was induced by a single intraperitoneal injection of 60 mg/kg body weight streptozotocin(STZ) (Sigma-Aldrich; Merck Millipore). Rats with blood glucose levels ≥ 250 mg/dl 24 h after STZ injection and which remained hyperglycemic for four days were classified as diabetic. A total of 25 diabetic rats were treated as diabetes group, and 25 normal rats without any treatment were used as the normal group. The rats in each group were randomized (*N* =5) and sacrificed after FFA examination on the 8^th^ day, at the 4^th^ week, 6^th^ week, 8^th^ week, and 10^th^ week after induction, respectively.

### 2.3. Animal Examination of Fluorescein Fundus Angiography (FFA)

Fluorescein fundus angiography (FFA) is one of the common and main methods for diagnosing DR. The rats in each group were treated with FFA (Heidelberg Spectraalis HRA, Heidelberg, Germany) to examine the right eye of rats. The rats were given intraperitoneal injection of sodium pentobarbital (40 mg/kg) for anesthesia after being weighed, pupil dilation was performed with one drop of compound topicamide (Mydrin-P○R; Santen, Osaka, Japan) and local anesthesia with alcaine, and then the corneal surface was coated with methyl cellulose to keep moist. During FFA examination, SD rats were intraperitoneally injected with 10% sodium fluorescein injection (0.001 ml/g, International Medication Systems, Dunstable, United Kingdom) for quick examination.

### 2.4. Experimental Samples

In order to label all blood-circulating vessels, an intravascular perfusion of fluorescent tomato lectin was performed [11]. Anesthetized SD rats were intravenously injected with 100 *μ*l fluorescein isothiocyanate-conjugated tomato lectin (1 mg/ml; Sigma-Aldrich; Merck Millipore) and 500 *μ*l fluorescein isothiocyanate (1 mg/ml; Yuanye Bio-Technology; Shanghai; China). Tomato lectin bound uniformly to the luminal surface of endothelial cells [12] and labeled all blood vessels with adequate blood supply. At 15 mins after injection, rats were perfused with stroke-physiological saline solution for 5 mins through the left ventricle at pressures of 80-120 mmHg under anesthesia. The vitreous humor and retina were isolated from the eyes under an ×2.5 anatomic microscope. The vitreous humor was isolated for VEGF-A ELISA and the retina for hematoxylin and eosin (H&E) staining, periodic acid-Schiff (PAS) staining, fluorescence imaging techniques, and expression of proinflammatory proteins on the 8^th^ day, at the 4^th^ week, 6^th^ week, 8^th^ week, and 10^th^ week after induction of DM, respectively.

### 2.5. Estimation of VEGF-A in Vitreous Humor

Isolated vitreous humor was homogenized in 185 *μ*l sterile phosphate buffer saline (PBS) after being frozen at -80°C for 5 min. According to the manufacturer's instructions, a rat VEGF-A ELISA kit (RayBiotech Inc, Norcross, GA, USA) capable of detecting both VEGF-A isoforms (RayBiotech Inc, Norcross, GA, USA) was used to estimate the level of VEGF-A proteins in the vitreous homogenate. The antibodies in the kit have >95% crossreactivity with rat.

### 2.6. H&E-Stained Retinal Preparations

One-fourth of the retinal tissue in each sample was isolated from normal and diabetic rats and fixed in 4% paraformaldehyde solution at 20°C for 2 h. Samples were subsequently sectioned (5 *μ*m), stained with H&E, and examined under a light microscope (magnification, ×400; Zeiss AG, Oberkochen, Germany) to determine the area and number of RGCs present in the samples.

### 2.7. Periodic Acid-Schiff Stain- (PAS-) Stained Retinal Preparations

One-fourth of the retinal tissue in each sample was isolated from each group and fixed in 4% paraformaldehyde solution for at 20°C for 24 h. Samples were subsequently placed into trypsin fluid for 40 mins at 37°C, stained with PAS, and examined under a light microscope (magnification, ×400; Zeiss AG) to calculate the number of acellular strands and the ratio of endotheliocytes to pericytes (E/P).

### 2.8. Fluorescence Imaging Techniques for Flat Retinal Preparations

Retinal flat mounts immersing in marker solutions were processed to visualize the vascular basement membrane. Prior to immersion staining, one-fourth of the retinal tissue in each sample was incubated for 45 mins at room temperature in 5% normal bovine serum in PBS containing 0.5% Triton-X-100 (0.5% T-PBS). Subsequently, the flat mounts were incubated overnight at 4°C in a marker solution containing rabbit polyclonal anti-type IV collagen antibody solution (1 : 300; ab19808; Abcam, Cambridge, UK) for basement membrane [9]. Fluorescent goat anti-rabbit immunoglobulin (Ig) G (1 : 45; BA1105; Wuhan Boster Biological Technology, Ltd., Wuhan, China) was treated as a secondary antibody. Subsequent to secondary incubation at 20°C for 5 mins, the retinal flat mounts were washed three times in 0.5% T-PBS, kept into DAPI for 5 mins, and washed another three times in 0.5% T-PBS. Then, the retinal flat mounts were prepared in a Vectashield (Wuhan Boster Biological Technology, Ltd.) and analyzed using a Zeiss LSM 710 confocal laser scanning microscope to determine the area and number of retinal neurocytes and the number of type-IV collagen strands.

### 2.9. Quantitative Real-Time PCR Analyses for VEGF and Various Inflammation-Related Molecules of Retina

In order to measure the mRNA expression levels of VEGF, IL-6, CD18, ICAM, and TNF-*α* in retinal tissue, total RNA was isolated from the one quarter of the retinal tissue remains using an extraction reagent (TRIzol; Invitrogen, Carlsbad, CA) and reverse-transcribed with a HiFiScript cDNA Synthesis Kit (First-Strand, CoWin Biosciences, china). PCR was performed using TaqDNA polymerase (Servicebio®, WuHan, China) in a thermal controller (Gene Amp PCR system; Applied Biosystems, Foster, CA). The primer sequences (Sangon Biotech, ShangHai, China) are as follows: 5′-GAGGCCGAAGTCTGTTTG-3′ (forward primer) and 5′-GGTTTGTCGTGTTTCTGGA-3′ (reverse primer) for VEGF, 5′-CACCAGGAACGAAAGTCAA-3′ (forward primer) and 5′-CAACAACATCAGTCCCAAGA-3′ (reverse primer) for IL-6, 5′-CAGCAGAAGGACGGAAAC-3′ (forward primer) and 5′-GGAGGAGGACACCAATCA-3′ (reverse primer) for CD-18, 5′-CCAGCCCCTAATCTGACCT-3′ (forward primer) and 5′-CTAAAGGCACGGCACTTGT-3′ (reverse primer) for ICAM, 5′-CAGCCAGGAGGGAGAAC-3′ (forward primer) and 5′-GTATGAGAGGGACGGAACC-3′ (reverse primer) for TNF-*α*, and 5′-AGCCATGTACGTAGCCATCC-3′ (forward primer) and 5′-ACCCTCATAGATGGGCACAG-3′ (reverse primer) for *β*-actin, respectively.

### 2.10. Image Processing and Statistical Analysis

IPP 6.0 and ImageJ 2.0 were used to process images, and IBM SPSS 19.0 (IBM Corporation, Armonk, NY, USA) statistical software was used for statistical analysis of the obtained data. Two-way ANOVA was conducted for multiple mean values, and independent sample *t*-test was conducted for data between groups. All data were treated with mean ± standard deviation. *P* < 0.05 was statistically significant.

## 3. Results

### 3.1. Metabolic Condition of Rats

**Figure 2:**
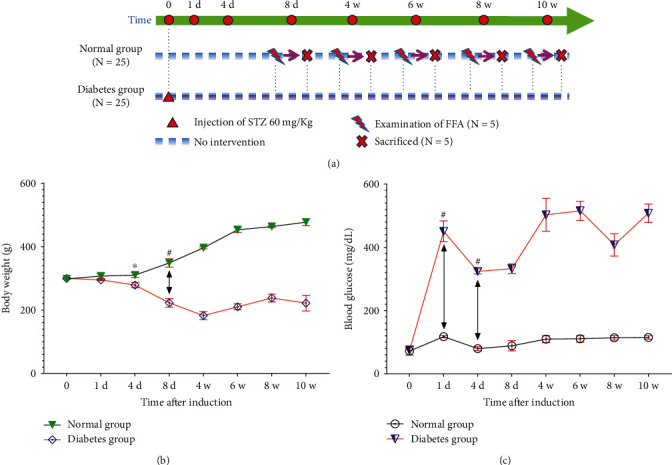
Changes in body weight and blood glucose levels of SD rats in this study. (a) Experimental design scheme. Twenty-five SD rats were intraperitoneally injected with 60 mg/kg STZ and treated as the diabetes group, and 25 normal SD rats were used as the normal group. Body weight (b) and blood glucose levels (c) of animals in this study. Data presented with mean ± standard deviation (^∗^*P* < 0.05, normal group vs. diabetes group on 4^th^ day; ^**#**^*P* < 0.01, normal group vs. diabetes group on 1^th^ day, and 4^th^ day, 8^th^ day, respectively). Abbreviations: d: day; w: week; *N*: number; STZ: streptozotocin; FFA: fluorescein fundus angiography.

The metabolism of rats was shown in Figure 2 after intraperitoneal injection of STZ. On the 4^th^ day after DM induction, the body weight of SD rats in the diabetes group was reduced compared with the normal group (*P* < 0.05), and with the progress of disease, the body weight of SD rats in the diabetes group was significantly lower than that in the normal group (*P* < 0.01). In addition, the blood glucose level of SD rats in the diabetes group increased significantly 24 hours after intraperitoneal injection of STZ, which was significantly different from that in the normal group (*P* < 0.01), and the difference remained significant throughout the 10-week observation period after DM induction.

### 3.2. Animal Examination of FFA

**Figure 3:**
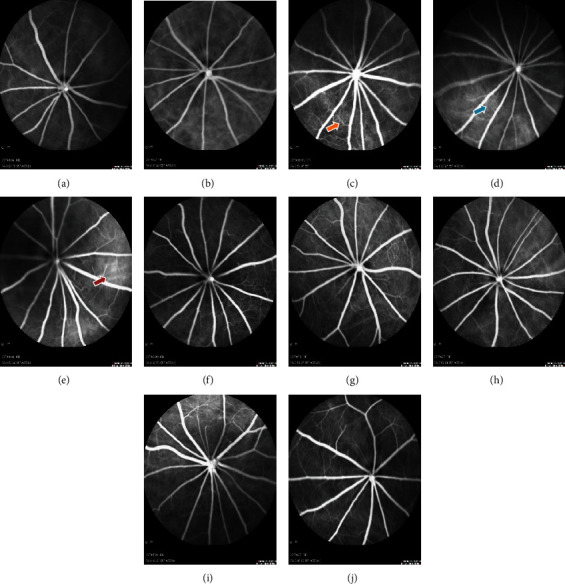
Morphological changes of retina in FFA. (a)–(e) were FFA examination images of diabetic rats on the 8^th^ day, at the 4^th^ week, 6^th^ week, 8^th^ week, and 10^th^ week after induction in the diabetes group, respectively, while (f)–(j) represented FFA images in the normal group at corresponding time points. Yellow arrow indicated vascular tortuosity and dilation, blue arrow indicated rough and leakage of vascular, and red arrow indicated typical leaky vessels with vascular tortuosity and dilation. Scale bar: 200 *μ*m.

In order to observe the changes of the retinal tissue vascular network system in diabetic SD rats during the 10-week observation period, FFA examination was performed (Figure 3). The optic disc of SD rats was located in the center of the retina, and the retinal blood vessels were radiated. After intraperitoneal injection of sodium fluorescein in SD rats for 3-5 s, retinal arteries began to fill, retinal vein laminar flow was observed for 5-7 s, and retinal vessel fluorescence decreased significantly for 3-6 mins until the fluorescence disappeared completely. The whole skin of SD rats was yellow at the end of examination. During the 10-week observation period after induction of DM, the SD rats in the diabetes group showed obvious vascular tortuosity and dilation at the 6^th^ week, and the peripheral roughness and leakage began to appear at the 8^th^ week, while the obvious vascular leakage and dilation appeared at the 10^th^ week. However, no such phenomenon was observed in the normal group during the 10-week observation period after induction.

### 3.3. Retinal H&E Staining

**Figure 4:**
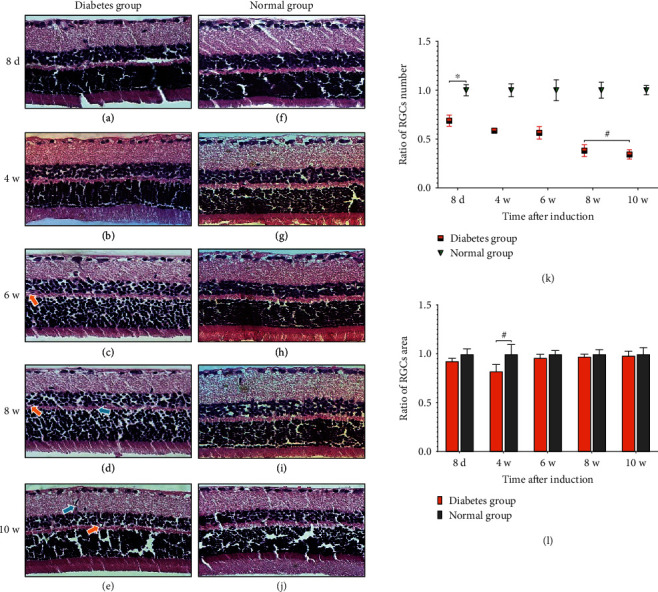
The number and area of RGCs and the morphological changes in two groups of retinal tissue HE staining, respectively (magnification, ×400). Retinal tissue looseness and edema and loosely arranged cells were observed in the diabetic group but not in the normal group. (a)–(e) were the HE staining of the diabetes group.(f)–(j) represented HE staining in the normal group at corresponding time points. (k, l) represented the ratio of RGCs and RGCs area between the two groups at each time, respectively (^∗^*P* < 0.01, ^**#**^*P* > 0.05). Blue arrows indicated new vessel buds, and yellow arrows indicated abnormally dilated microvessels. Scale bar: 25 *μ*m. Abbreviations: d: day; w: week; RGCs: retinal ganglion cells.

In this study, the morphological changes between the retinal nerve fiber layer and the outer nuclear layer of rats were observed on the 8^th^ day, at the 4^th^ week, 6^th^ week, 8^th^ week, and 10^th^ week after DM induction, respectively, and the number and area of RGCs were counted and measured in each group (Figure 4). The number of RGCs in SD rats in the diabetes group decreased significantly compared with that in the normal group (*P* < 0.01) on the 8^th^ day after induction, and with the progression of the disease, the number of RGCs in the diabetes group decreased gradually. However, there was no significant difference in the number of RGCs in the diabetes group at the 8^th^ week and 10^th^ week (*P* > 0.05), and the number of RGCs tended to be stable. Furthermore, there was no significant difference in retinal ganglion cell area between the diabetes group and the normal group during the 10-weeek observation period after induction (*P* > 0.05). Microvascular dilatation was observed at the 6^th^ week, and the formation of new vessel bud and obvious microvascular dilatation was observed at the 8^th^ week, while typical new vessel bud was observed between the ganglion cell layer and the inner nuclear layer at the 10^th^ week.

### 3.4. Retinal PAS Staining

**Figure 5:**
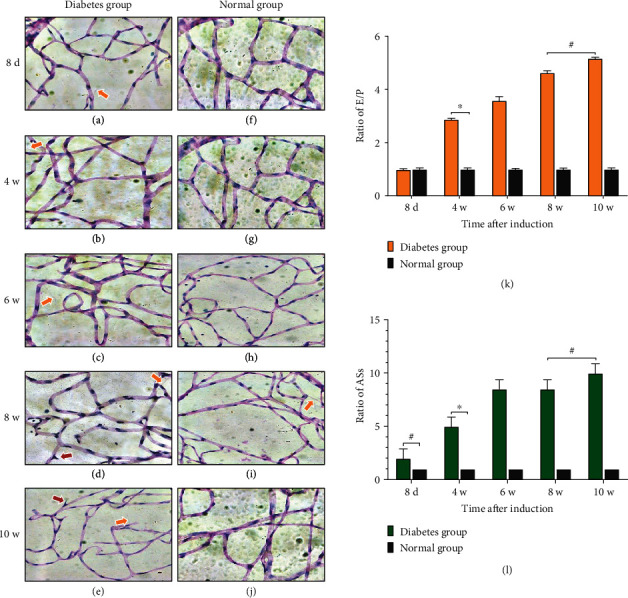
The ratio of E/P and number of acellular strands and the morphological changes between the two groups in PAS staining of retinal tissue (magnification, ×400). (a)–(e) were the PAS staining of the diabetes group. (f)–(j) represented PAS staining in the normal group at corresponding time points. (k, l) represented the ratio of E/P and acellular strands of rats in two groups at each time, respectively. (^∗^*P* < 0.01, ^**#**^*P* > 0.05). Yellow arrows indicated acellular strands, and red arrows indicated new vessel buds. Scale bar: 25 *μ*m. Abbreviations: d: day; w: week; E/P: endotheliocytes to pericytes; ASs: acellular strands.

In the PAS staining, the nuclei of endotheliocytes were oval, and the dye was pale, while the nuclei of pericytes were circular and the dye was darker than the endotheliocytes (Figure 5). E/P ratio and acellular strands of retinal tissue vessels of SD rats in each group were counted on the 8^th^ day, at the 4^th^ week, 6^th^ week, 8^th^ week, and 10^th^ week after DM induction, respectively. The E/P ratio in retinal tissue of SD rats in the diabetes group began to be significantly higher than that in the normal group at the 4^th^ week (*P* < 0.01), and with the progress of disease, a significant difference was maintained between the two groups. The number of acellular capillaries was increased on the 8^th^ day after induction in the diabetes group, and the trend of the increase was worse with the progress of the disease course. The significant difference began to appear at the 4^th^ week after DM induction (*P* < 0.01). At 8^th^ week, new vessel buds and a large number of acellular strands were observed, while typical new vessel buds and a large number of acellular filaments were also observed at the 10^th^ week.

### 3.5. Estimation of VEGF-A in Vitreous Humor

**Figure 6:**
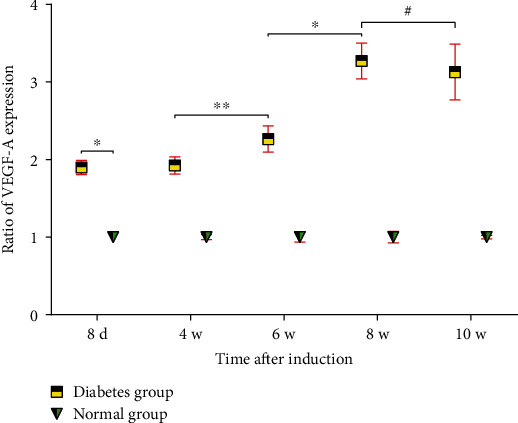
The ratio of the VEGF-A expression in vitreous cavity of SD rats in the diabetes group and normal group (^∗^*P* < 0.01, ^∗∗^*P* < 0.05, ^**#**^*P* > 0.05).

In this study, VEGF-A ELISA kit was used to detect the concentration of VEGF-A in vitreous cavity of SD rats, and the changes in the concentration of VEGF-A in vitreous cavity of rats in each group were determined on the 8^th^ day, at the 4^th^ week, 6^th^ week, 8^th^ week, and 10^th^ week after successful modeling (Figure 6), respectively. On the 8^th^ day after DM induction, the concentration of VEGF-A in vitreous cavity of SD rats in the diabetes group became higher than that in the normal group at the same time point (*P* < 0.01) and remained at a high concentration with the progression of the disease. However, the VEGF-A concentration in vitreous cavity of SD rats in diabetes group began to increase further at the 6^th^ week after the successful modeling, reaching the peak at the 8^th^ week.

### 3.6. Fluorescence Imaging Techniques for Retinal–Flat Preparations

**Figure 7:**
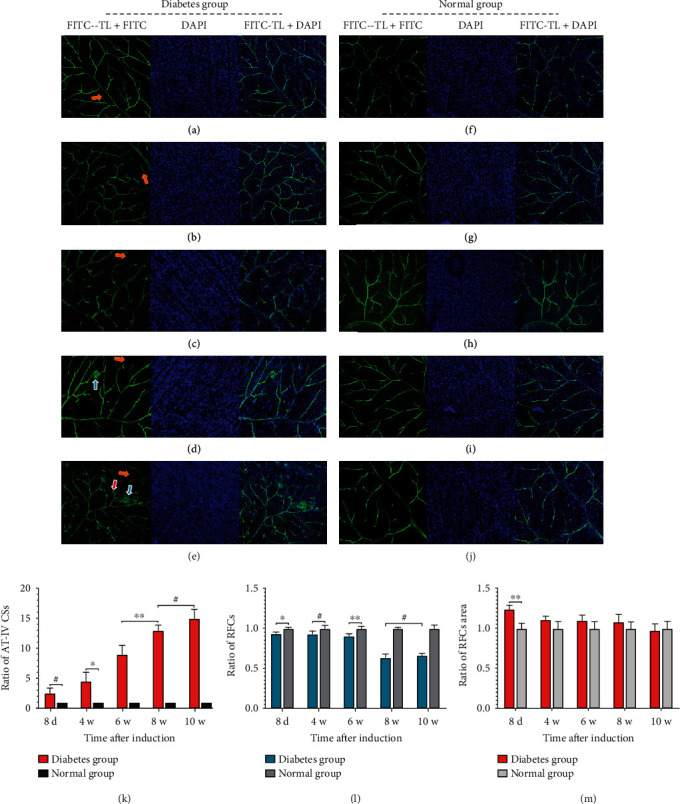
(a)–(e) were the immunohistochemical imaging of the diabetes group and (f)–(j) in the normal group, respectively (magnification, ×100). (k)–(m) represented the ratios of type IV collagen-positive strands, retinal cells, and the area of retinal cells in the diabetes group and the normal group at each time, respectively. Yellow arrows indicated type IV_+_ collagen-positive strands, blue arrows indicated vascular permeability, and red arrows indicated new vessel buds. (^∗^*P* < 0.05, ^∗∗^*P* < 0.01, ^**#**^*P* > 0.05). Scale bar: 75 *μ*m. Abbreviations: d: day; w: week; FITC: fluorescein isothiocyanate; TL: tomato lectin; AT-IV CSs: anti-type IV collagen strands; RFCs: retinal flat cells.

In this study, the changes of microvessels and cells in the retinal tissue of SD rats were observed by fluorescein isothiocyanate (FITC)-tomato lectin, rabbit polyclonal anti-type IV collagen antibody, and DAPI-labeled retinal-flat (Figure 7). Compared with the normal group, the number of anti-IV_**+**_ collagen strands crosslinked between retinal vessels in the diabetes group began to increase on the 8^th^ day after induction of DM. It was higher than that in the normal group at the 4^th^ week with statistical difference (*P* < 0.05), and with the progression of disease, there was a significant statistical difference (*P* < 0.01). The number of nerve cells in the retinal tissue decreased in the diabetes group compared with that in the normal group on the 8^th^ day after induction (*P* < 0.05), but there was no statistical difference at the 4^th^ week (*P* > 0.05). With the progress of the disease course, the number of nerve cells in retinal tissue decreased significantly in diabetes group at the 6^th^ week (*P* < 0.01) and became worse at the 8^th^ and 10^th^ week, but the number of cells tended to be stable. In addition, the retinal tissue cell area of SD rats in the diabetes group was significantly higher than that in the normal group (*P* < 0.01) on the 8^th^ day, but there was no statistical difference between the two groups with the progression of disease (*P* > 0.05). We observed that the retinal tissue vessels of SD rats in the diabetes group showed obvious tortuosity and local leakage at the 8^th^ week, and the conditions were aggravated, and the formation of new vessel buds were observed at the 10^th^ week.

### 3.7. The mRNA Expression Levels of VEGF, IL-6, CD18, ICAM, and TNF-*α* in Retinal Tissue

**Figure 8:**
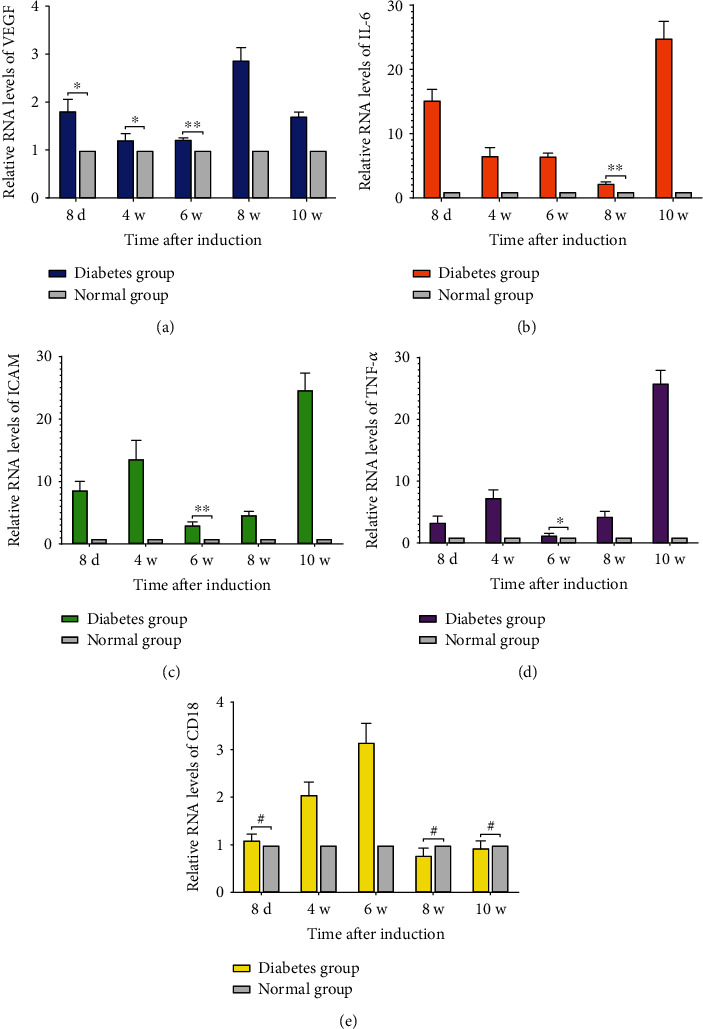
(a)–(e) represented the ratio of the VEGF RNA expression, the ratio of the IL-6 RNA expression, the ratio of the ICAM RNA expression, the ratio of the TNF-*α* RNA expression, and the ratio of the CD18 RNA expression in retinal tissues, respectively (^∗^*P* < 0.05, ^∗∗^*P* < 0.01, ^**#**^*P* > 0.05).

Many molecules with inflammatory characteristics were detected in the retina of diabetic animals (Figure 8). In this study, we found that the mRNA expression of VEGF in the retinal tissue of SD rats in the diabetes group began to increase on the 8^th^ day after induction, which was higher than that in the normal group (*P* < 0.05), and reached the peak at the 8^th^ week. During the 10-week observation period, the mRNA expression of VEGF in the retinal tissue maintained a state of high expression in the diabetes group. At the same time, the mRNA expression of IL-6, ICAM, and TNF-*α* in retinal tissue began to be highly expressed in the diabetes group on the 8^th^ day after induction, and the difference was statistically significant compared with that in the normal group at the same time point (*P* < 0.01). With the progression of the disease, the mRNA expression levels of IL-6, ICAM, and TNF-*α* were in a high expression state in the diabetes group, and a new peak appeared at the 10^th^ week. However, the CD18 mRNA expression in the retinal tissue was significantly higher in the diabetes group than that in the normal group from the 4^th^ week after model establishment (*P* < 0.01) and reached the peak at the 6^th^ week after induction.

## 4. Discussion

**Figure 9:**
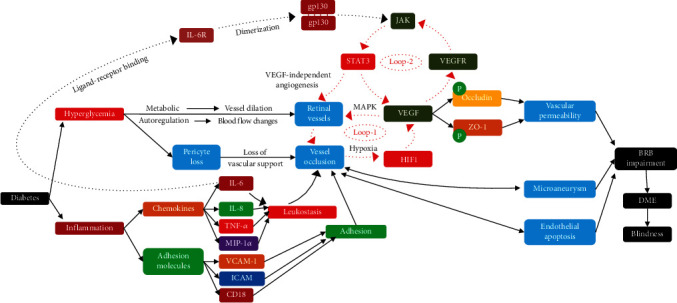
The pathogenetic process and molecular mechanism of the early occurrence and development of DR. There are two loops in the mechanism of DR: loop-1 (microcirculation obstruction-hypoxia-HIF1-VEGF-neovascularization-microcirculation obstruction) and loop-2 (VEGF-VEGFR-JAK-STAT3-VEGF).

Diabetic retinopathy affects up to 90% of patients with diabetes, with 5% progressing to legal blindness. It has become increasingly clear that diabetic retinopathy affects not only retinal vasculature and retinal neuronal and glial cells but also a variety of inflammatory mediators [4, 5, 13–17] (Figure 9). In our study, a series of RGC changes were observed during the 10-week observation period. The loss of RGCs could lead to a variety of degenerative diseases, including DR and glaucoma [18]. RGCs showed increased apoptosis during the fourth week of the diabetes course [6] and neuronal dysfunction across all retinal layers 12 weeks after STZ-induced diabetes in rats, which is consistent with a study [19] of the late functional loss [20]. As diabetes progresses, RGCs began to decrease at the 6^th^ week [21, 22]. However, previous reports [23–25] suggested that the ganglion cells underwent apoptosis after 12 weeks of STZ-induced diabetes. Based on the observation of different nodes and interspecies difference between our study and previous reports [6, 20, 23–25], oxidative stress, and the high expression of inflammatory mediator precursors caused by hyperglycemia, the apoptosis of RGCs increased on the 8^th^ day after induction. Although the molecular mechanisms of cell depletion or structural abnormalities are not clear, inflammation, oxidative stress, or advanced glycation end products have been suggested to be responsible for pathologic changes in the retina, including a decrease in the number of RGCs [22, 26–28]. Furthermore, a report suggested that the size of RGCs was unchanged compared with the control group during the three-month observation period [25], which is consistent with our observations, except on the 8th day after induction. We speculated that the oxidative stress and the release of inflammatory mediator might cause abnormal metabolism in the retinal neuronal cells in the early stage of diabetes, thereby leading to the increase of the area of the retinal neuronal cells on the 8^th^ day after induction.

The morphological changes seen in retinal microvessels of DR include many pathological changes, such as early loss of pericytes, loss of endothelial cells, increased vascular permeability, and capillary dropout [29, 30]. Our study suggested that the ratio of E/P, acellular strands, and type IV collagen-positive strands in the diabetes group were significantly higher than those in the normal group at the 4^th^ week after induction, while the vascular permeability and vessel buds were observed at the 8^th^ week and 10^th^ week after induction. These predict the abnormal structure of retinal vessels and the occurrence of new blood vessels, which will lead to retinal new vessels. The earliest identified lesion in the diabetic retina is pericyte loss [7, 31]. Pericyte loss progresses over time to endothelial cell loss, resulting in the formation of acellular capillaries [32]. However, the mechanism of pericyte loss in early DR is unclear. Akagi et al. [33] proposed that this mechanism was related to the sorbitol pathway, because they found that aldose reductase was present in human retinal capillary pericytes through immunohistochemical staining, but not in endothelial cells. A study [34] pointed out that hyperglycemia or galactosemia can cause abnormal secretion or function of platelet-derived growth factor B chain, which may selectively affect pericyte activity and lead to pericyte apoptosis. The loss of pericytes in the vascular wall can lead to the increase of E/P ratio, which will lead to the formation of microhemangioma.

In our study, we also found that the vitreous VEGF-A concentration and the expression level of VEGF RNA in the retinal tissue began to show high expression on the 8^th^ day after induction in the diabetes group and reached the peak at the 8^th^ week. Ayalasomayajula et al. [8] found that the concentration of VEGF and the VEGF mRNA expression in retinal tissue of diabetic rats were significantly higher than those in the normal control group on the 8^th^ day after induction, which was consistent with our results. In the 4-week or 12-week observation period, the VEGF concentration and the VEGF mRNA expression in retinal tissue of diabetic rats at the 4^th^ week were the highest compared with the normal control group (*P* < 0.01) [9, 35]. However, the VEGF-A concentration in vitreous cavity and the VEGF mRNA expression level in retinal tissue at the 8^th^ week in our study were different, which might be due to the sharp upregulation of the VEGF-A concentration and the VEGF mRNA expression level in diabetic rats caused by temporary acute high blood glucose concentration, ischemia, and hypoxia in the diabetic group. In addition, the overexpression of VEGF is related to altered angiogenesis and the increases of retinal vascular permeability, resulting in retinal dysfunction [10]. Based on the above studies, the occurrence of new vessel buds or new vessels may be inevitable, and the increasing vascular permeability seems to be a natural development of the DR progression. These new blood vessels may lead to retinal microaneurysms or pathological neovascularization. One study demonstrated that the number of type IV_+_ collagen strands without any evidences of endothelial proliferation and containing no cellular elements had been the earliest morphological changes at the 1^th^ and 4^th^ week after DM induction [9]. The type IV_+_ strands indicate a potential relationship between the vascular degeneration and the very early stages of DM [36]. Meanwhile, the vascular regression maybe represent hollow vessels or structurally-collapsed without blood flow, resulting in “vascular ghosts” [37]. Thus, the presence of type IV_+_ collagen strands in vascular degeneration is a residue of vascular basement membrane composition. These results suggest that endothelial cell degeneration and vascular basement membrane residue caused by diabetes are due to vascular degenerative changes.

Many reports have indicated that retinal microvascular injury is linked to upregulation of several cytokines such as IL-6, TNF-*α*, VEGF, and CD18 and the pathological overexpression of intercellular and vascular cell adhesion molecules (ICAM-1 and vascular cellular adhesion molecule-1) [38–41]. Our study finds that the mRNA expression levels of IL-6, ICAM, and TNF-*α* were always high in the diabetes group during the 10-week observation period, and the high expression levels began to appear on the 8^th^ day after the DM induction, but the mRNA expression level of CD18 began to increase significantly at the 4^th^ week after induction, and reached the peak at the 6^th^ week. Increasing evidence suggests that the IL-6 signaling pathway plays a prominent role in the endothelial cell dysfunction and vascular inflammation of DR [42–45]. A report [46] has found that the levels of TNF-*α* and IL-6 in the diabetes group were significantly higher than those of the normal group at the 2^th^ week after induction, while another study [47] has suggested that IL-6 and TNF-*α* became significantly elevated in diabetic retina after STZ injection as compared with normal rats at the 4^th^ week after induction and continued to the 8^th^ week. Our results are similar to what have been reported in the two reports. In addition, the expression of CD18 in retina became increased at the 1-week old diabetic rats [48]. Elevated levels of CD18 in neutrophils were present in each stage of DR: the more severe the disease, the higher the levels are [49]. The leukostasis is known to be increased in retinal blood vessels in diabetes, and this process is mediated via ICAM-1, while the retinal ICAM-1 levels were significantly increase when compared with the nondiabetic control group after 1 week of diabetes [50]. ICAM-1 is upregulated by several stimuli, including VEGF, poly (ADP-ribose) polymerase activation, oxidative stress, and dyslipidemia [51–53]. To our knowledge, the dynamics of these factors are attributable to metabolic disorders and the activation of inflammatory responses caused by hyperglycemia during diabetes. Our study is a continuation of the above studies and bears similarities. At the same time, it tries to offer an indepth and detailed understanding of the pathological changes in the early stage of DR.

In conclusion, our study indicated the abnormal alterations of microvessels, microstructure, and inflammatory mediators at the early stage of DR, which confirms and supplements the previous research, and also promotes an indepth understanding and exploration of the pathophysiology and underlying pathogenesis of DR. The results are sufficient to warrant further investigations, offer new insight into the pathogenesis of diabetic retinopathy, and offer novel targets to inhibit the ocular disease.

## Data Availability

The datasets used and/or analyzed during this study are available from the corresponding author on reasonable request.
